# Characterization of a gold coated cantilever surface for biosensing applications

**DOI:** 10.1140/epjti/s40485-014-0011-5

**Published:** 2015-02-21

**Authors:** Ann-Lauriene Haag, Yoshihiko Nagai, R Bruce Lennox, Peter Grütter

**Affiliations:** Department of Physics, McGill University, 3600 Rue University, Montreal, QC H3A 2T8 Canada; Research Institute of the McGill University Health Centre, 2155 Guy Street, Montreal, QC H3H 2R9 Canada; Department of Chemistry and FQRNT Centre for Self Assembled Chemical Structures, McGill University, 801 Sherbrooke Street West, Montreal, QC H3A 2K6 Canada

**Keywords:** Surface stress, Cantilever sensing, Biosensor, Oligonucleotide, Electrochemistry

## Abstract

Cantilever based sensors are a promising tool for a very diverse spectrum of biological sensors. They have been used for the detection of proteins, DNA, antigens, bacteria viruses and many other biologically relevant targets. Although cantilever sensing has been described for over 20 years, there are still no viable commercial cantilever-based sensing products on the market. Several reasons can be found for this – a lack of detailed understanding of the origin of signals being an important one. As a consequence application-relevant issues such as shelf life and robust protocols distinguishing targets from false responses have received very little attention.

Here, we will discuss a cantilever sensing platform combined with an electrochemical system. The detected surface stress signal is modulated by applying a square wave potential to a gold coated cantilever. The square wave potential induces adsorption and desorption onto the gold electrode surface as well as possible structural changes of the target and probe molecules on the cantilever surface resulting in a measurable surface stress change. What sets this approach apart from regular cantilever sensing is that the quantification and identification of observed signals due to target-probe interactions are not only a function of stress value (i.e. amplitude), but also of the temporal evolution of the stress response as a function of the rate and magnitude of the applied potential change, and the limits of the potential change.

This paper will discuss three issues that play an important role in future successful applications of cantilever-based sensing. First, we will discuss what is required to achieve a large surface stress signal to improve sensitivity. Second, a mechanism to achieve an optimal probe density is described that improves the signal-to-noise ratio and response times of the sensor. Lastly, lifetime and long term measurements are discussed.

## Introduction

Nanomechanical structures can be used for label-free and low-cost biosensors that offer high sensitivities. In recent years, several nano and micromechanical structures have been described as possible biosensor platforms, such as nanomechanical cantilevers [1-4], resonators [5,6], and optomechanical structures [7]. The most common detection principles due to biological binding effects are changes in surface stress [8,9] and mass [10,11].

Here we focus on a cantilever sensing platform that detects changes in surface stress. In our platform, a cantilever is coated with a gold layer that serves two purposes. First, this gold layer is used as a support structure of probe molecules bound to the surface typically using thiol linkers; this in principle gives the sensor specificity [12]. What is often not considered is the second role of this gold layer, as it can act as a very sensitive transducer that is located within nanometers of the probe molecules that sense the biological binding events [9,13]. In our system, the surface potential of the gold coated cantilever is controlled and changed over time to induce changes of the surface coverage of the adsorbing ion. Changing the presence of any ionic or charged species near the surface leads to a large change of surface stress. This is based on the well established fact that surface stress is directly proportional to the surface charge density [14]. Surface concentration changes of charged species can be induced by applying an electrochemical potential which generates conformational changes of probe molecules.

Our approach to increasing the dynamic range of the stress signal is to drive the adsorption and desorption of ions to the cantilever surface, thus inducing a large measurable and characteristic surface stress change [15]. This movement of ions can be modulated as a function of time, allowing signal averaging techniques to be used. If clean gold surfaces are used, the resultant reproducible time dependent stress signals include information on the target-probe system, such as ion diffusion times and polymer dynamics. This information can be used for biochemical sensors or in fundamental studies (e.g. for the investigation of the folding dynamics of proteins). Reliable signal and thus target identification can be based on recognition of the complex time dependent stress patterns in addition to the information given by signal amplitudes.

In our experiments, we change the presence of ions near the surface by combining a conventional cantilever stress sensing system with a standard three-electrode electrochemical system. All experiments are performed in buffer solution with the cantilever acting as a working electrode (WE), a platinum wire as the counter electrode (CE) and a Ag/AgCl (sat. KCl) electrode as the reference electrode (RE). The electrodes are connected through a potentiostat allowing a voltage to be applied between the working (cantilever) and the reference electrode thus measuring the current flowing between the working and the counter electrode (voltammetry) [16]. Upon application of a square wave potential to the gold coated cantilever between +/− 200 mV, chloride ions that are present in solution will ad-/desorb on the surface which leads to a change in surface charge density [17] and therefore to a change in surface stress [14]. In our system the stress-induced bending of the cantilever is measured by optical beam deflection methods and translated into a quantitative surface stress signal by using Stoney’s formula [18] and appropriate calibrations [19,20].

The electrochemical aspect of our sensor system serves two distinct purposes. First, it is used to clean and electrochemically characterize the surface of the gold coated cantilever. Secondly, it is used to apply a controlled, time dependent potential to the cantilever to induce repetitive surface stress changes. This first point is very important, as surface stress and surface stress changes are driven by surface charge density, which is a function of the cleanliness of a system [9]. Recalling that a clean metallic surface typically takes about 1 microsecond to be contaminated in air by absorbable organic molecules – hence the need for ultra high vacuum conditions (UHV) to investigate surface phenomena. Electrochemistry allows a systematic cleaning and characterization of surfaces in solution. Note that compared to the concentration of rest gas (‘contaminations’) in UHV, solutions are very seldom as pure – clean solution to background contaminations would need to be at a level of 1 part in 10^13^ to achieve similar lifetimes of clean surfaces in solution as in UHV.

**Figure 1:**
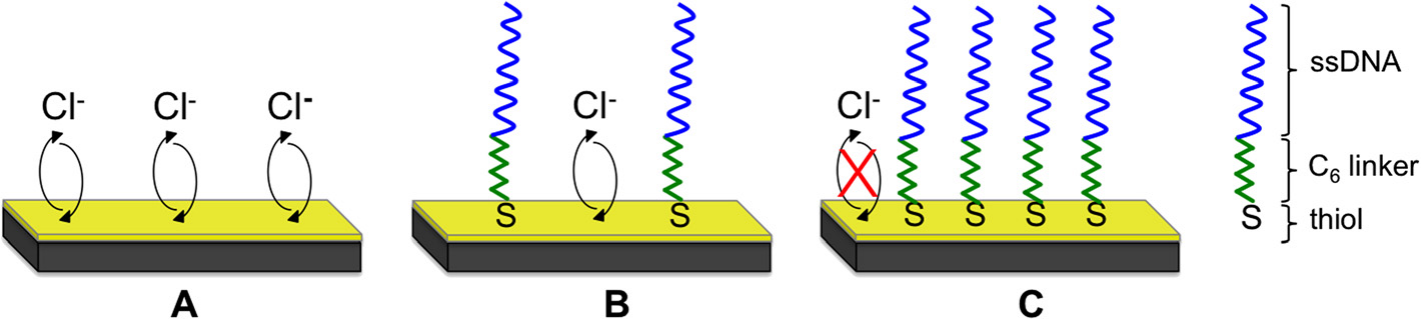
**Potential induced adsorption/desorption of chloride ions on different functionalized gold surfaces.** Schematics describe the relationship between surface stress change and available surface area. **A**, a bare and clean gold surface is shown. Chloride ions can freely interact with the whole surface and large surface stress changes are measured. Upon binding of some single-stranded oligonucleotide to the surface **B**, fewer ions can react with the surface leading to a smaller surface stress change. Once the layer is densely packed on the surface **C**, the surface stress change vanishes as no ions can reach the surface anymore.

An important insight is that the surface stress change on the cantilever is proportional to the available and accessible gold surface area. This can be used to measure and optimize the concentration of probes on a cantilever that leads to a decrease in the available gold surface area due to the target molecules covering part of the gold surface (Figure 1) [9]. On a clean gold coated surface, a large number of ions can interact with the surface resulting in a large surface stress change signal. If part of the surface is covered by molecules, in this case thiolated single-stranded oligonucleotide, fewer ions can access the surface leading to a smaller surface stress change. Covering the surface complete densely with a monolayer of molecules will hinder the ions to reach the surface and no large change in surface stress is observed. Residual (much smaller) stress changes can be due to steric hindrance and other effects. Our group has previously shown surface stress changes for aptamer functionalized gold surfaces. The aptamer undergoes a conformation change into a more compact state upon binding to its cognate ligand therefore increasing the available gold area, i.e. increased surface stress, compared to its relaxed state [15].

In this paper we will discuss three issues that most nanomechanical based sensors are facing and present protocols to improve these issues. This will be the foundation for possible applications of biosensors applicable to real-life samples containing not only the analyte of interest, but many background ‘contaminants’. These three challenges are: 1. How can large surface stress signals be achieved? Any contaminants on the surface will reduce the available surface area and therefore lead to smaller surface stress change. We will present an electrochemical cleaning protocol that results in a clean gold surface, leading to a large and quantitatively reproducible surface stress signal when surface charge densities change. 2. How can the signal-to-noise ratio be improved? An optimal probe density is required for good signal-to-noise ratios of the surface stress change. This is achieved by using a multi-step functionalization protocol recently described by Nagai et al. [15]. 3. How can sensors with long term stability and realistic shelf life be manufactured? For device applications one needs to know how the sensors perform during long term measurements in the analyte of interest. We will present long term measurements of our cantilever platform in solution and discuss our observations.

## Results and discussion

### Electrochemical cleaning

We have tested different cleaning procedures by quantifying surface cleanliness electrochemically *in situ* and using surface science techniques *ex situ*. Based on results published by Fischer et al. [21], we tested the following cleaning protocols: 1. Electrochemical sweep of the gold coated cantilever in 50 mM potassium hydroxide (KOH) from −0.2 to 1.2 V (vs. Ag/AgCl (sat. KCl)), 2. Electrochemical sweep in 50 mM potassium perchlorate (KClO_4_) from −0.8 to 1.4 V (vs. Ag/AgCl (sat. KCl)) and 3. Piranha solution (Three parts concentrated sulfuric acid (H_2_SO_4_) and one part hydrogen peroxide (H_2_O_2_); note that great caution is necessary when using piranha solution) treatment of the cantilever for 5 min. We find that the KClO_4_ and KOH- mediated processes result in the cleanest surface as monitored using *ex situ* X-ray photoelectron spectroscopy (XPS) and *in situ* cyclic voltammetry. The atomic percent surface composition measured from XPS results of the survey scan for the piranha cleaning method results in 40.2% gold, 33.6% carbon and 26.1% oxygen. A huge improvement of these value, i.e. higher gold percentage can be seen for the KOH sweep as well as the KClO_4_ sweep. For KOH the composition is 65.3% gold, 30.0% carbon and 4.74% oxygen, which is very comparable to the values for the KClO_4_ sweep with 61.7% gold, 33.8% carbon and 4.5% oxygen. The KClO_4_ sweep is chosen to be the primary cleaning step for all further experiments, as this is a standard media for electrochemical cleaning of gold. Additionally, perchlorate has a very small affinity for gold and will not adsorb onto the gold surface.

**Figure 2:**
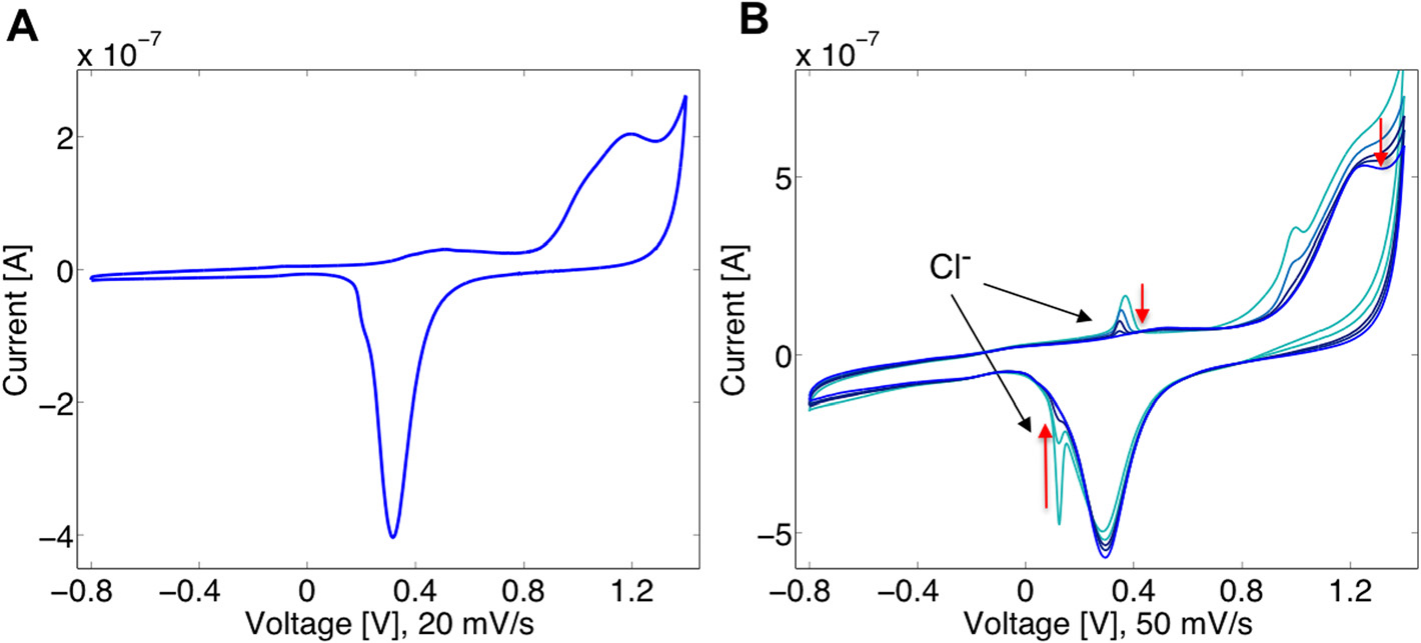
**Electrochemical cleaning protocol on gold coated cantilever in 50 mM KClO**_**4**_ **.** Cyclic voltammogram in 50 mM KClO_4_ (vs. Ag/AgCl (sat. KCl)). **A**, the standard gold spectra is shown at 20 mV/sec indicating a clean gold surface. **B**, a chloride contaminated surface is electrochemically cleaned. After six full potential sweeps, the chloride peaks vanish and a clear distinct gold reduction peak is observed.

In detail, with this method the cantilever is electrochemically cleaned in 50 mM KClO_4_ by sweeping the applied potential in solution from −0.8 to 1.4 V (vs. Ag/AgCl (sat. KCl)). Sweeping the potential from −0.8 to 1.4 V oxidizes the gold surface, whereas the reverse step, going from 1.4 to −0.8 V reduces the resulting gold oxide. This cleaning step serves to intrinsically remove contaminants from the surface. The cyclic voltammogram (current vs. potential, CV) scan is performed at 20 mV/sec and is continuously repeated until a reproducible CV of gold is achieved, indicating the removal of any contaminates. In Figure 2A, a CV from a bare clean gold evaporated cantilever is shown. Multiple peaks that overlap are observed between 0.9-1.2 V, which correspond to the oxidation of gold. A significant sharp reduction peak is observed at 0.35 V. Figure 2B shows a cleaning process in action. The CV spectrum has two additional peaks at 0.35 V and 0.15 V consistent with chloride on the gold surface. Over the course of six full potential cycles, the chloride redox peaks vanish, leaving a clean bare gold surface.

**Figure 3:**
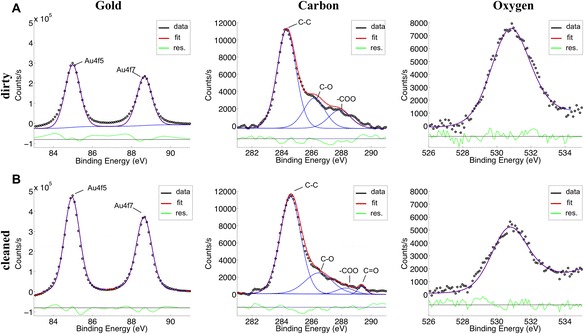
**Uncleaned and cleaned gold coated samples to test effectiveness of electrochemical cleaning protocol with XPS.** X-Ray Photoelectron Spectroscopy (XPS) data are shown for the Au4f, C1s and O1s peaks for uncleaned **(A)** and cleaned **(B)** gold samples. The percent surface composition for the uncleaned gold surface is 43.7% gold, 41.2% carbon and 9.4% oxygen based on the survey scan. The gold peak can be increased to 61.7% by electrochemically cleaning the surface. Additionally, carbon and oxygen are decreased to 33.8% and 4.5% respectively.

To further verify the effectiveness of the chosen cleaning protocol, X-ray photoelectron spectroscopy (K-Alpha X-Ray Photoelectron Spectrometer system, Thermo Scientific, USA) was performed on evaporated gold surface on a silicon substrate. In Figure 3, the individual high-resolution spectra of Au4f, C1s and O1s are shown for two different samples: (A) gold sample that has not been cleaned, and (B) an electrochemically cleaned gold sample. Both samples are made from a piece of a silicon wafer with a thermally evaporated 2 nm titanium adhesion layer followed by thermal deposition of 100 nm gold. The samples were stored under ambient condition for 1 week. Prior to the experiment, one sample is rinsed with MilliQ water and blow-dried using a nitrogen stream (uncleaned), the other sample is electrochemically cleaned using 50 mM KClO_4_ and dried with nitrogen (cleaned). The atomic percent surface composition of the uncleaned sample is measured to be 43.7% gold, 41.2% carbon and 9.4% oxygen based on the survey scan. In Figure 3B, the electrochemically cleaned sample is shown. The time between the cleaning and measuring the sample with XPS was about 30 min. The overall composition of the surface was 61.7% gold, 33.8% carbon and 4.5% oxygen. Compared to the dirty sample, a relative increase of about 40% was observed for the gold peak and a relative decrease of 20% and 50% for the carbon and oxygen peak contamination was observed. Higher measured levels of gold on the surface means there is less contamination surface, demonstrating the effectiveness of the electrochemical cleaning protocol. The remaining oxygen and carbon peaks result from exposing the sample to air for 30 min prior to measuring the surface composition and cannot be avoided. This was verified by sputter cleaning a gold sample in UHV until a clean Auger spectrum was acquired, then exposing it to air for 20 minutes. The surface composition measured by the survey scan resulted in 66.1% gold, 31.8% carbon and 2.2% oxygen.

**Figure 4:**
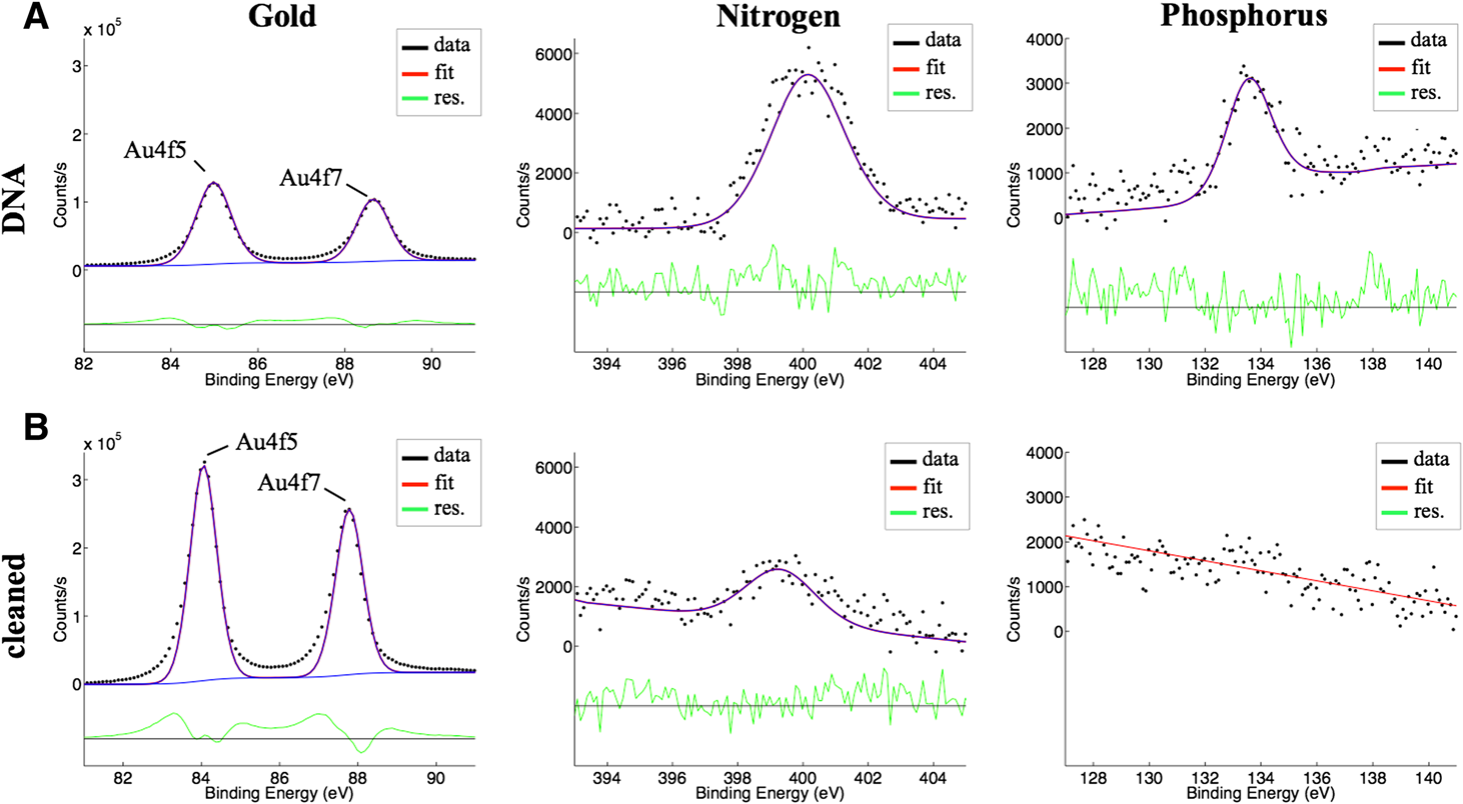
**XPS measurements of the electrochemical removal of an oligonucleotide functionalization layer on the gold sample.** X-Ray Photoelectron Spectroscopy (XPS) data are shown for the Au4f, N1s and P2p peaks for oligonucleotide functionalized **(A)** and electrochemical cleaned **(B)** gold samples. A clear increase in the gold peak as well as a clear removal of the phosphate peak of the cleaned sample can be seen.

An important feature of the intrinsic electrochemical cleaning protocol is the ability to revert the sensor surface to its base state in situ by removing the oligonucleotide functionalization layer. A cantilever that is functionalized with 10 μM 25-mer thiolated oligonucleotide was measured with XPS and the % at composition for gold, nitrogen and phosphorus is 11.1%, 11.64% and 5.97%. A clear phosphorus peak in the XPS spectra indicates successful oligonucleotide functionalization, see Figure 4A. Subsequently, a sample functionalized under the same conditions is electrochemically cleaned with 50 mM KClO_4_ to remove the oligonucleotide. XPS of the oligonucleotide functionalized and electrochemically cleaned sample is shown in Figure 4B. The % at composition for gold, nitrogen and phosphorus is measured as 45.86%, 9.73% and 0% (not measurable). The clear removal of the phosphorous peak indicated the removal of the oligonucleotide functionalization layer from the surface. Subsequently, a more thorough electrochemical cleaning can be done.

### Surface stress measurements

A large signal-to-noise ratio can be achieved by precisely controlling the probe density on the surface. If the surface is completely covered with the molecule of interest, ions cannot interact with the surface, and only very small surface stress changes are measured. Additionally, a complete coverage of the surface by probe molecules is detrimental to a fast response time of the sensor, as many target molecules will not be able to interact with the probe molecules. Therefore, it is crucial to achieve an optimal probe density to maximize the signal-to-noise ratio of the measurements. Our group has developed a multistep oligonucleotide functionalization protocol that enables for systematic control of the functionalization density and thus leads to high quality sensor functionalization with good reproducibility’s. After the electrochemical cantilever cleaning described above, a surface stress pattern is recorded for 30 min during application of a square wave potential between +/− 200 mV with a 10 min period. Afterwards, the cantilever is incubated in a 10 μM thiolated single stranded oligonucleotide solution for 5 min and another surface stress pattern is recorded. This step is repeated until the desired coverage is achieved. This can be controlled by first monitoring the decrease in surface stress amplitude due to the increased probe coverage and therefore a decreased availability in gold area and then evaluating the surface stress pattern change due to comparative adsorption of chloride ions in solution and the negatively charged oligonucleotide phosphate backbone [22]. The effective density of the oligonucleotide layer can be determined by using 12-ferrocenyl-1-dodecanethiol (Fc(CH_2_)_12_SH) to label unfunctionalized areas of the gold surface. The net area associated with unfunctionalized gold is determined from the integrated area of the electrochemically active ferrocene label. This process was previously shown by Nagai et al. [15], details are described below.

**Figure 5:**
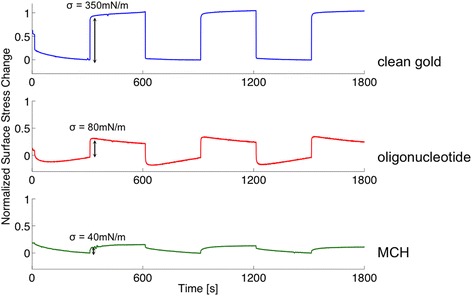
**Surface stress change on a clean compared to functionalized gold coated cantilevers.** Surface stress change pattern of the cantilever as a response to an applied square wave potential at +/− 200 mV with a 10 min period. The blue trace shows the response of a clean gold evaporated surface measuring a large surface stress change of σ = 350 mN/m. Upon functionalization with single-stranded oligonucleotide (red trace), the surface stress change decreases to σ = 80 mN/m. Covering the surface with 6-mercapto-1-hexanol (MCH) (green trace) leads to a low surface stress change of σ = 40 mN/m.

From an applications point of view achieving a reproducible sensor response is highly desirable. In our system this translates into the necessity of achieving a reproducible probe surface coverage. The surface probe density can be characterized by measuring the chloride-induced stress changes of the cantilever (all experiments are performed in Tris–HCl 10 mM NaCl 50 mM pH 7.4 buffer (TN buffer)). To drive adsorption and desorption of chloride ions to the cantilever gold surface, a square-wave potential is switched between −200 and +200 mV, with a 10 min period. As a result of the square wave potential, the cantilever will undergo characteristic bending due to the induced surface stress change. In Figure 5, the surface stress change patterns for a gold surface that is clean (blue), partially functionalized with single stranded thiolated oligonucleotide (red) and 6-mercapto-1-hexanol (MCH) (green) versus time are shown. These three cases demonstrate the relationship between surface stress change and available gold surface area very well. The surface stress change for clean gold results in a large signal with an amplitude of σ = 350 mN/m. Furthermore, it shows a response pattern that is in phase and similar in shape to the applied square wave potential. This is because chloride ions are essentially immediately driven to/from the surface; surface charge density (leading to a change in surface stress) thus follows the profile of the applied potential.

Upon functionalization of the cantilever gold surface with 25-mer thiolated single stranded oligonucleotide with a packing density of roughly 9% as described in [15], the surface stress amplitude decreases and the response pattern starts to deviate from that of the applied square potential. The trace shows an upward slope trend for regions where +200 mV is applied and a downward slope for regions where −200 mV is applied. The amplitude decreases from 350 mN/m to 80 mN/m compared to the clean gold surface. This supports the fact that if less gold surface is available for the chloride ions to adsorb to, the smaller the surface stress change will be. The oligonucleotide covers a part of the surface and makes it less accessible for chloride ions to adsorb/desorb at the applied potentials. The change in shape of the response curve is due to changes in the structure of single-stranded oligonucleotide when potential is applied and results from interactions between the charged oligonucleotide phosphate backbone and the gold surface and hydration shell dynamics. Quantitative modeling of these various phenomena and their interplay is presently being investigated. What is clear at this stage is that the detailed shape of the stress response curve allows determination of the probe surface coverage.

To further demonstrate this principle, another sample was functionalized with MCH, a short thiolated C_6_ linker for 5 min. MCH binds strongly to the gold surface resulting in a densely packed layer. Ion adsorption is blocked and the capacitance of the electrode is reduced [23]. The surface stress change pattern shows an even stronger deviation from the shape of the applied potential and a further decrease in stress amplitude to values such as 40 mN/m. Note that the pattern change provides a potentially more robust signal to detect hybridization than the amplitude, which can vary depending on the number and type of defects in the self-assembled monolayer [24].

In summary, we point out two key observations. This first is that the amplitude of the surface stress change at the switching potential decreases, the more the gold surface is blocked by any molecules, allowing fewer chloride ions to ad-/desorb onto the surface. The absolute value of the amplitude is a function of the initial gold cleanliness. Contaminants in the solution that competitively bind to the clean gold surface (e.g. bromide in a chloride solution) and potential-induced conformational changes in the probe molecules also affect the signal amplitude. Reproducible large absolute signal values can be achieved by suitable gold cleaning protocols as described above. Note that the average absolute surface stress value is 280 mN with a reproducibility of 40%.

The second key point is that the surface stress pattern is characteristic of the nature of the surface bound molecules (either of the probe functionalization layer or the probe-target complex). The former can be used to (re-)generate well defined probe functionalization layers *in situ*. The latter allows for the determination of the presence of target molecules as recently demonstrated by Nagai et al. [15], who reported on the pattern shape change due to oligonucleotide hybridization and aptamer-protein interactions of optimized sensing layers. The change in the pattern can be attributed to the negatively charged phosphate backbone of the DNA and mechanical property changes upon hybridization. A negative applied potential will repel the DNA from the surface leaving the DNA in a standing up position [25]. Over time, a double layer will build up that screens the DNA charge which results in a relaxation of the DNA into its neutral state. This is reflected in the slope change in the surface stress change pattern observed at −200 mV. At positive potentials, the DNA is attracted to the gold surface and is lying down. The relaxation of this position is visible in the slope change of the surface stress change pattern. In passing, we note that by varying the temporal period of the applied potential, conformation dynamics can be studied, potentially allowing label free fundamental experimental insights into important topics such as protein folding.

### Long-term measurements

**Figure 6:**
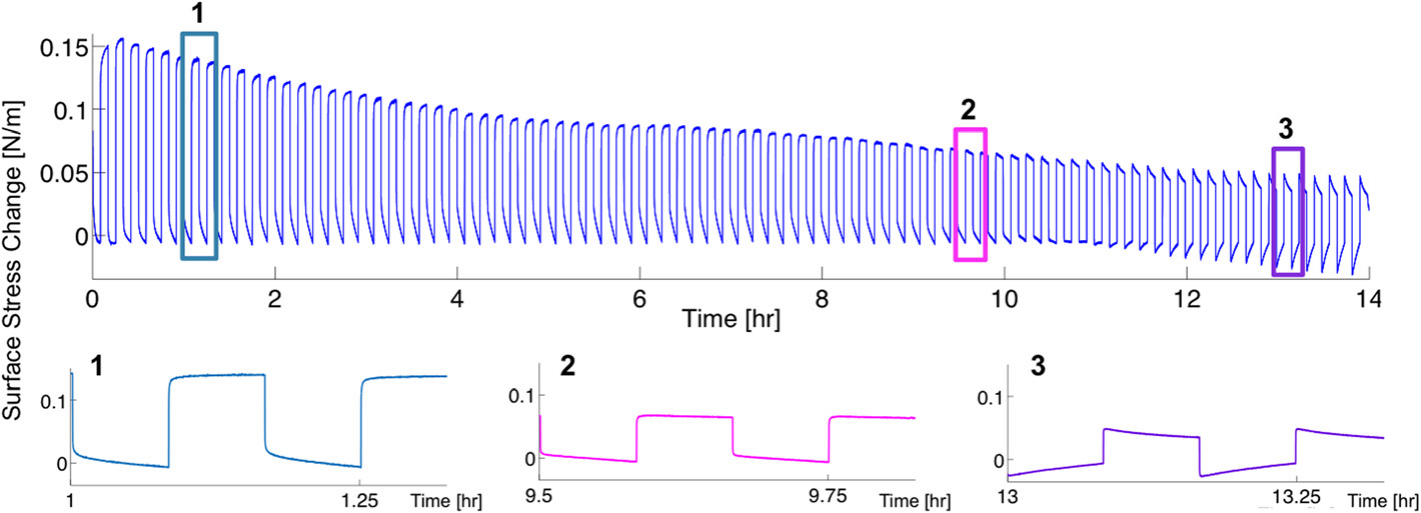
**Longterm surface stress measurement of gold coated cantilever in buffer.** Longterm surface stress of a gold coated cantilever in TN buffer recorded for 14 hrs. A square wave potential between +/− 200 mV with a period of 10 min was applied to the cantilever. A zoom of three sections are shown and labeled as 1, 2 and 3. At (1) a large surface stress change is measured. After around 9.5 hours (2), the pattern starts to change resulting in a negative slope for +200 mV and a positive slope for −200 mV, clearly visible after 13 hrs (3). Furthermore, the overall surface stress change amplitude decreases indicating that that ions chemisorb onto the surface over time covering part of the gold surface.

An important question is how long the sensor response remains stable. Because cantilever based sensors are very sensitive, their response is expected to drift and change as a function of time, concentration of contaminants, etc. We have performed long-term stability recordings in the TN buffer used for our oligonucleotide and aptamer protein measurements. The cantilever was electrochemically cleaned in 50 mM KClO_4_, rinsed with MilliQ water and placed in buffer. No oligonucleotide functionalization was performed. A square wave potential between +/− 200 mV with a period of 10 min is applied to the cantilever for 14 hrs (84 cycles). The surface stress change of the cantilever was recorded over time, as shown in Figure 6. Overall, the surface stress amplitude decreases from 150 mN/m to 100 mN/m after 4 hrs, to 60 mN/m after 10 hrs, and finally less than 50 mN/m after 14 hrs. Additionally, a pattern change is observed. The slope of the pattern at +200 mV changes from a positive to a negative trend after 8 hrs. A similar change is observed for the slope at −200 mV changing from a negative to a positive trend after 11 hrs. A zoom into three different regions of the curve after 1, 9.5 and 13 hrs is shown in the Figure 6. The overall decrease in amplitude is attributed to chemisorbed ions on the surface leading to a decrease in the available gold area. A CV was recorded in 50 mM KClO_4_ before and after the long-term measurement. The charge increases by 85%, indicating an increase in the capacitance, assuming that the active area has remained constant. The experiment starts by applying a potential of −200 mV and ends 14 hours later at +200 mV after 84 cycles. Generally, one observes that the sensor remains stable for 10 hrs in TN buffer before competitive chemisorption takes place leading to an unreliable measurement. This is likely a function of the purity of the buffer components and water used to prepare the buffer solution. With the intrinsic electrochemical cleaning set-up, the cantilever can be restored to its original state, i.e. a clean gold surface *in situ*.

## Conclusion

We have addressed and discussed three issues that are fundamental to successful cantilever biosensor integration and relevant for many other sensor platforms and applications. First, in sensors where the signal relies on surface charge changes such as in chemFETS or cantilever based sensors, a clean chemical functionalization layer support surface is crucial in order to obtain large signals. Here we report an electrochemical cleaning method of the gold surface often used to support the thiolated probe molecules by sweeping a potential that is applied to the sensor surface between −0.8 and 1.4 V in 50 mM KClO_4_ until a reproducible cyclic voltammogram is obtained. XPS data verifies the effectiveness of this cleaning method. The advantage of this method is that the sensor can be cleaned intrinsically without the use of any harsh chemicals that might harm the sensor integration environment. This cleaning step will remove the functionalization layer of the sensor restoring it to its original state. Second, we demonstrate how to achieve a high signal-to-noise ratio by carefully controlling the probe coverage of the sensor. A multistep functionalization protocol is described relying on characteristic changes in the stress response as a function of probe density to *in situ* electrochemical stimulation. This systematically provides a higher quality layer and a better control of the surface coverage, leading to higher signal-to-noise ratios and to a reproducible, predictable sensor response. Surface stress change measurements on a clean gold surface, oligonucleotide and MCH modified gold cantilever surfaces are described. In all experiments described here, a square wave potential between +/− 200 mV is applied to the cantilever in TN buffer. These experiments confirm that the surface stress change is proportional to the available gold area. Additionally, the surface stress change pattern gives detailed information about conformational changes on the surface upon applying a potential. Lastly, long term stability measurements are shown in buffer indicating the sensor lifetime to be about 10 hrs. The origin of this limitation is currently being investigated. After 10 hrs, a electrochemical cleaning step is necessary to recover the surface to its initial high sensitivity state.

## Methods

### Oligonucleotide preparation

All experiments were performed using a 25-mer thiolated single stranded oligonucleotide with a sequence of 5′-HS-SC6- TCGGATCTCACAGAATGGGATGGGC-3′ (by IDT Technology, USA). The stock oligonucleotide solution was prepared by diluting to a concentration of 100 μM by in 40 μl of TE buffer (Tris–HCl 10 mM, 5 mM EDTA, pH 7.4). Prior to each experiment, the oligonucleotide is desalted by incubating in 25 mM TCEP (Fisher Scientific, USA) for 1 hr followed by a subsequent purification step using a NAP-5 column (GE Healthcare, UK). The desalting step breaks of the disulfide bond of the oligonucleotide making it reactive to the gold surface. The final oligonucleotide concentration for all experiment was 10 μM.

### Cantilever preparation

Silicon cantilevers (CSC38/tipless/no Al-coating, Mikromash) are solvent cleaned with acetone, isopropanol and methanol, the cantilevers before thermal evaporation. A 2 nm thick titanium adhesion layer is evaporated onto the cantilever with a rate of 0.9 Å/s followed by 100 nm of gold at a rate of 1 Å/s at a pressure of < 3×10^−6^ mBar and stored under ambient condition before use. To define the gold area that is exposed to the electrochemical set-up, a thin layer of apiezon wax (Apiezon wax W, APWK, USA) that is dissolved in trichloroethylene (TCE) (Fisher Scientific, USA) is applied to the base of the cantilever leaving an area of 1.0 mm^2^ exposed.

### Electrochemical cleaning

Argon is injected into potassium perchlorate (50 mM KClO_4_) to remove any oxygen in solution. Subsequently, prior to each experiment the cantilever is electrochemically cleaned in 50 mM KClO_4_ (Fisher Scientific, USA) by cycling the potential between −0.8 to 1.4 V at 20 mV/sec until a repeatable gold cyclic voltammogram peak is observed (CHI 1000, CH Instruments, USA). The cantilever is set up as the working electrode, a platinum wire (1 mm thick, Alfa Aesar, USA) is used as the counter electrode and a standard Ag/AgCl (sat. KCl) reference electrode (BASi, USA).

## Abbreviations

UHV: Ultra high vacuum
DNA: Deoxyribonucleic acid
CV: Cyclic voltammetry
TN buffer: Tris–HCl 10 mM NaCl 50 mM pH 7.4 tris(hydroxymethyl)aminomethane hydrogen chloride natrium chloride
XPS: X-ray photoelectron spectroscopy
SAM: Self-assembled monolayer
MCH: 6-mercapto-1-hexanol
